# COVID‐19 in a country with a very high prevalence of diabetes: The impact of admission hyperglycaemia on mortality

**DOI:** 10.1002/edm2.279

**Published:** 2021-06-14

**Authors:** Carlos Martínez‐Murillo, Christian Ramos Peñafiel, Lourdes Basurto, Lourdes Balcázar‐Hernández, Karen Pellón, Eder Flores López, Beatriz Li Gómez, Mercedes Estefania Ledesma, Rodrigo Rivera Tapia, Elizabeth Madera Maldonado, Monica Bejarano Rosales, Gilberto Barranco Lampon, Juan Francisco Zazueta

**Affiliations:** ^1^ Hematology Department of Hospital General de México, Dr Eduardo Liceaga México City Mexico; ^2^ Hospital Regional de Alta Especialidad Ixtapaluca Ixtapaluca Mexico; ^3^ Endocrine Research Unit Centro Medico Nacional Siglo XXI IMSS Mexico City Mexico; ^4^ Endocrinology Department, Hospital de Especialidades Centro Medico Nacional Siglo XXI IMSS Mexico City Mexico; ^5^ Facultad de Medicina UNAM, SECISS Mexico City Mexico; ^6^ Hospital General Cuautitlán, Gral, José Vicente Villada Cuatitlán México

**Keywords:** COVID‐19, diabetes, hyperglycaemia, mortality

## Abstract

**Aims:**

To evaluate the frequency of diabetes and admission hyperglycaemia in Mexican COVID‐19 patients, to describe the clinical and biochemical characteristics of patients with admission hyperglycaemia and to determinate the impact of diabetes and admission hyperglycaemia on COVID‐19 severity and mortality.

**Methods:**

A multicentric study was performed in 480 hospitalized patients with COVID‐19. Clinical and biochemical characteristics were evaluated in patients with admission hyperglycaemia and compared with non‐hyperglycaemic patients. The effect of diabetes and admission hyperglycaemia on severity and risk of death were evaluated.

**Results:**

Age was 50.7 ± 13.6 years; 68.3% were male. Some 48.5% (*n* = 233) had admission hyperglycaemia; 29% (*n* = 139) of these patients had pre‐existing diabetes. Patients with admission hyperglycaemia had more requirement of invasive mechanical ventilation (IMV), higher levels of urea, D‐dimer and neutrophil‐lymphocyte ratio (NLR), as well as lower lymphocyte count. An association between admission hyperglycaemia with IMV and D‐dimer with glucose was found. Age ≥50 years (OR 2.09; 95%CI 1.37–3.17), pre‐existing diabetes (OR 2.38; 95%CI 1.59–5.04) and admission hyperglycaemia (OR 8.24; 95%CI 4.74–14.32) were risk factors for mortality.

**Conclusions:**

Admission hyperglycaemia is presented in 48.5% of COVID‐19 patients. Diabetes and admission hyperglycaemia are associated with the severity of disease and mortality. This study shows the devastating conjunction of hyperglycaemia and COVID‐19.

**Clinical trial registration:** Clinical characteristics of patients with COVID‐19, DI/20/204/04/41 (Hospital General de Mexico) and NR‐13‐2020 (Hospital Regional de Alta Especialidad Ixtapaluca).

## Graphical abstract

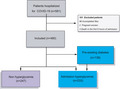

**Novelty and impact statement**

‐ Diabetes and admission hyperglycaemia are risk factors in severity and mortality among COVID‐19 patients. Patients with pre‐existent diabetes and hyperglycaemia showed an OR 8.24 (95%CI 4.74–14.32) for mortality.
‐ The results of present study denote the devastating conjunction of two pandemics, diabetes and COVID‐19, in a country with a very high prevalence of metabolic diseases.
‐ Early detection of hyperglycaemia in patients with COVID‐19, both with and without diabetes, timely treatment and the restoration of normoglycaemia are essential.

## 1 INTRODUCTION

Coronavirus disease 2019 (COVID‐19) is caused by a novel coronavirus named Severe Acute Respiratory Syndrome Coronavirus 2 (SARS‐CoV‐2).^1^ The highest proportion of severe cases occurs in adults ≥60 years of age, and in patients with diabetes, hypertension, obesity, chronic obstructive pulmonary disease, and cardiovascular, renal, and cerebrovascular diseases.^1^, ^2^, ^3^ The SARS‐CoV‐2 accesses host cells via the binding of its spike glycoprotein to angiotensin‐converting enzyme 2 (ACE2), sialic acid receptor, transmembrane serine protease 2 (TMPRSS2), extracellular matrix metalloproteinase inducer (CD147), cathepsin B and L; all of these factors are expressed in endothelial cells. Endothelial dysfunction is a determinant of COVID‐19.^4^ The main complications of COVID‐19 include acute respiratory distress syndrome (ARDS), cardiac injury, liver dysfunction, acute kidney injury, bacteraemia, diffuse intravascular coagulation and hyperglycaemia. Hyperglycaemia is common in patients with COVID‐19, predominantly in severe cases.^3^

Hyperglycaemia in hospitalized patients, irrespective of its cause, is associated with adverse outcomes.^3^ Hyperglycaemia could be present in patients with known or undiagnosed diabetes, or during acute illness (termed ‘stress hyperglycaemia’).^5^

Stress hyperglycaemia has two scenarios: patients with undiagnosed diabetes or impaired glucose tolerance, and patients who develop hyperglycaemia as a result of the severe stress and increased counterregulatory hormones.^6^

Diabetes and hyperglycaemia are associated with a poor outcome (higher severity and mortality) in patients with COVID‐19.^7^ Admission hyperglycaemia has been observed as a predictor of radiographic imaging of SARS‐CoV2, regardless of previous diabetes diagnosis.^8^

Diabetes is one of the most common causes of morbidity and mortality around the world.^9^ In 2019, 463 million people had diabetes, with an expectation that this number will rise to 578 million people worldwide by 2030 and 700 million by 2045.^9^ In the top 10 countries with more cases of diabetes, Mexico ranks 6th worldwide, with 12.8 million people affected.^9^

The specific mechanisms of hyperglycaemia in COVID‐19 are not clear.^10^ A relation between SARS‐CoV‐2 and ACE2 receptor, expressed in the liver and in the endocrine pancreas, has been proposed.^10^ Hepatocytes and pancreatic beta cells could be infected by SARS‐CoV‐2 through glycosylated ACE2 receptor, promoting the development of insulin resistance and impaired insulin secretion, inducing hyperglycaemia.^10^ The impairment in β‐cell insulin secretion may worsen pre‐existing diabetes or determine the appearance of hyperglycaemia in cases of non‐diabetes.^10^ A vicious circle has been proposed: SARS‐CoV‐2 decreases insulin secretion and promotes the appearance/worsening of insulin resistance, inducing hyperglycaemia, which, in turn, may further damage β‐cells, with a worsening of insulin resistance.^11^

In low‐ and middle‐income countries, there is little information about the frequency of hyperglycaemia in COVID‐19 patients and its impact on outcome severity. In our population, there is a high rate of diabetes, hypertension, overweight and obesity, which are risk factors for severe disease in SARS‐CoV‐2 infection.^12^ A study in Mexican population has shown that diabetes, particularly early‐onset diabetes, is a risk factor for COVID‐19 lethality and mortality; however, the effect of diabetes and hyperglycaemia on adverse outcomes in a clinical setting is not yet understood.^13^

The aims of our study were to evaluate the frequency of diabetes and admission hyperglycaemia in Mexican COVID‐19 patients, to evaluate the clinical and biochemicals characteristics of patients with admission hyperglycaemia and compare with non‐hyperglycaemia patients, and to determinate the impact of diabetes and admission hyperglycaemia on severity and mortality in a population of a low/middle‐income country.

## 2 SUBJECTS, MATERIALS AND METHODS

A cross‐sectional, retrospective, observational study was conducted in 480 patients hospitalized with COVID‐19 in three medical institutions of Mexico: General Hospital of Mexico Eduardo Liceaga, Regional Hospital of High Specialty Ixtapaluca and General Hospital of Cuatitlán Vicente Villada. These hospitals are responsible for the treatment of patients with COVID‐19, assigned by the government of Mexico. Patients were attended from March to July 2020.

### 2.1 Laboratory diagnosis of COVID‐19

SARS‐CoV‐2 testing was performed according to WHO recommendations.^14^ Samples from the upper respiratory tract (nasopharyngeal and oropharyngeal) were collected for SARS‐CoV‐2 testing by real‐time PCR. Only lower respiratory tract (expectorated sputum, endotracheal aspirate or bronchoalveolar lavage) samples were obtained in mechanically ventilated patients. Lung biopsies were not performed. SARS‐CoV‐2 testing was certified by the Institute of Epidemiological Diagnosis and Reference.^13^

**FIGURE 1:**
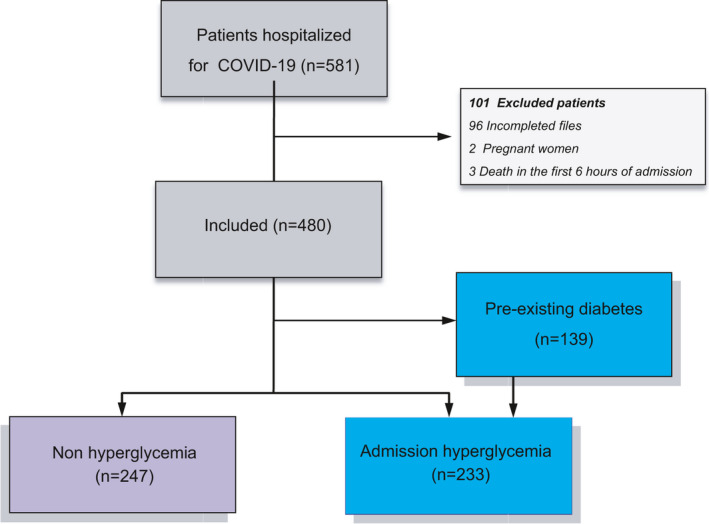
Flowchart of study population

Inclusion criteria were patients aged 18–80 years, clinical syndromes associated with SARS‐CoV‐2 infection that required hospitalization (mild and severe pneumonia, Acute Respiratory Distress Syndrome [ARDS], sepsis and sepsis shock),^14^ and positive SARS‐CoV‐2 testing (real‐time PCR). Pregnant women were not eligible for the study. Patients with incomplete data in medical records were excluded (Figure 1). All participating subjects were informed of the aim of the study and provided oral informed consent.

The clinical history, vital signs and laboratory data, obtained from electronic medical records, were analysed and evaluated. Information was collected on age, sex, morbidity and body mass index (BMI). The laboratory data collected were routine blood, blood glucose, lipid, liver and renal function, fibrinogen and D‐dimer levels.

COVID‐19 patients who had admission hyperglycaemia were compared against non‐hyperglycaemia COVID‐19 patients. Admission hyperglycaemia was defined as blood glucose levels ≥140 mg/dl occurring within a 24‐h period since the hospital admission.^5^, ^15^ Pre‐existing diabetes was confirmed by reviewing patients’ medical records. No patients had been receiving glucocorticoid treatment at the time of glucose level measurement. Date of death or required invasive mechanical ventilation (IMV) was available.

The diagnosis of pneumopathy was established with radiography chest plus thoracic ultrasonography or computed tomography.^16^

All patients were treated with standard protocol, including hydroxychloroquine, antiviral drugs or tocilizumab. Non‐critically ill patients with pre‐existing diabetes and glucose levels between 110 and 180 mg/dl continued with oral antidiabetic drugs. Sulphonylureas were discontinued in all cases. If glucose levels were above 180 mg/dl, an insulin regimen with basal‐bolus‐correction established. Critically ill patients and patients with hyperglycaemia on admission were treated with an insulin regimen and basal‐bolus‐correction (NPH, glargine and lispro).

The primary outcomes were presence of severe pneumonia, which was defined by the use of IMV and in‐hospital mortality.

### 2.2 Statistical analysis

The Kolmogorov‐Smirnov test was applied to determine the type of distribution of the continuous variables. Measures of central tendency and dispersion were calculated: mean ± standard deviation (SD) for parametric data or median and interquartile range (IQR) values for nonparametric data. Categorical variables were given as frequency rates and percentages. Mann‐Whitney U test or *t*‐test was used to compare quantitative measurements. In correlation analysis, Pearson correlation coefficient was used for the variables of normal distribution and Spearman correlation coefficient for those of skewed distribution.

The comparison of the proportion of the variables such as obesity, IMV and mortality was analysed with X^2^ test. Risk estimation was through the odds ratio and 95% confidence interval (OR, 95%CI). All the analyses were carried out with the statistical program Statistical Package for the Social Sciences (SPSS) version 20. *p *< .05 was considered significant.

## 3 RESULTS

### 3.1 Baseline characteristics of the patients with COVID‐19

A total of 480 patients were included. The mean age was 50.7 ± 13.6 years; 68.3% (*n* = 328) were male. Some 29% (*n* = 139) had pre‐existing diabetes, 32.3% overweight, 50.5% obesity and 24% hypertension. 48.5% (*n* = 233) of patients had admission hyperglycaemia. Demographic, clinical and biochemical characteristics of the patients are summarized in Table 1.

**TABLE 1:** Characteristics of COVID‐19 patients with admission hyperglycaemia and non‐hyperglycaemia

Variable	Total (*n* = 480)	Non‐hyperglycaemia (*n* = 247)	Admission hyperglycaemia (*n* = 233)	*p*‐value^a^
Age (years)	50.7 ± 13.6	48.9 ± 13.9	52.7 ± 13.0	.002
Sex				
Female (%)	31.7	27.5	36.1	.02
Male (%)	68.3	72.5	63.9	
Body Mass Index (Kg/m^2^)	29.9 ± 5.6	30.1 ± 5.8	29.7 ± 5.5	NS
Normal (%)	17.2	16.4	18	NS
Overweight (%)	32.3	34.3	30.2	
Obese (%)	50.5	49.3	51.8	
Hypertension (%)	24	19.4	28.8	.01
Invasive Mechanical Ventilation (%)	29	25.2	36	.009
Glucose (mg/dl)	120 (96–165.5)	98 (88–117)	166 (133–260)	.0001
Urea (mg/dl)	30.9 (17.8–48.2)	26.7 (15.9–46.4)	36 (20.1–56)	.001
Creatinine (mg/dl)	0.99 (0.78–1.3)	0.91 (0.7–1.2)	1.0 (1.3–8)	NS
Alanine aminotransferase (U/L)	41 (27.2–63.7)	41 (29–63.2)	39 (26–64.2)	NS
Aspartate aminotransferase (U/L)	43 (28–65)	45 (31–65.7)	41 (26–65)	NS
Neutrophil count (×10^9^/L)	7.56 (5.59–10.83)	7.22 (5–24–10.53)	8.1 (5.7–11.5)	NS
Lymphocyte count (×10^9^/L)	0.87 (0.60–1.2)	0.93 (0.63–1.3)	0.76 (0.5–1.06)	.001
NLR	8.81 (5.57–14.91)	8.36 (5.42–14.29)	9.4 (6.1–16.3)	.04
Platelet count (×10^9^/L)	210 (165–283.5)	210 (166.5–280.2)	205(165–284)	NS
Fibrinogen (mg/dl)	648.4 ± 210	647.8 ± 201.9	649.1 ± 219.9	NS
D‐dimer (ng/ml)	1111 (716.2–2296)	981.5 (670–1683)	1347 (868–3060.7)	.001

Note

Parametric variables are represented as mean ± standard deviation. Non‐parametric variables are represented as median and interquartile range (IQR).

Abbreviations: NLR: Neutrophil‐Lymphocyte Count Ratio; NS, No significant.

^a^ Differences between Non‐hyperglycaemia versus Admission hyperglycaemia.

### 3.2 Clinical and biochemical characteristics of COVID‐19 patients with admission hyperglycaemia

Of COVID‐19 patients with admission hyperglycaemia, 139 (59.6%) had pre‐existing diabetes. There were no patients with pre‐existing diabetes in non‐hyperglycaemia group. The clinical and biochemical characteristics of the COVID‐19 patients with admission hyperglycaemia and non‐hyperglycaemia were compared (Table 1). Age (48.9 ± 13.9 vs. 52.7 ± 13.0; *p *= .002), frequency of female sex (27.5% vs. 36.1%; *p *= .02) and hypertension (19.4 vs. 28.8%; *p *= 0.01) were higher in patients with admission hyperglycaemia. There were no significant differences with respect to BMI, obesity and overweight. Patients with admission hyperglycaemia were more likely to require IMV (25.2% vs. 36%; *p *= .009).

In the biochemical evaluation, patients with admission hyperglycaemia, compared with non‐hyperglycaemia patients, had higher levels of glucose [98 (88–117) vs. 166 (133–260) mg/dl; *p *= .00011], urea [26.7 (15.9–46.4) vs. 36.0 (20.1–56) mg/dl; *p *= .001], D‐dimer [981.5 (670–1683) vs. 1347.0 (868–3060.7) ng/ml; *p *= .001] and neutrophil/lymphocyte ratio (NLR) [8.36 (5.42–14.29) vs. 9.4 (6.1–16.3); *p *= 0.04], as well as lower lymphocyte count [0.93 (0.63–1.3) vs. 0.76 (0.5–1.06); *p *= 0.001]. Creatinine, transaminases and fibrinogen levels and platelet count did not show significant differences.

In a within‐group subset analysis of patients with admission hyperglycaemia, it was observed that women required IMV less frequently (Table 2). In contrast, it was found that patients of the IMV subgroup presented higher levels of urea, creatinine, neutrophil count and NLR. It was also observed that this group of patients presented a higher level of D‐dimer [1862 (1052–3908) vs. 1229 (766–2788) ng/ml; *p *= .001] and length of hospital stay [7 (4–13) vs. 5 (4–9) days; *p *= .03], compared to the subgroup of admission hyperglycaemia patients who did not require IMV.

**TABLE 2:** Characteristics of COVID‐19 patients with admission hyperglycaemia with and without Invasive Mechanical Ventilation (IMV)

	Without invasive mechanical ventilation (*n* = 153)	With invasive mechanical ventilation (*n* = 80)	*p*‐value
Age (years)	52.3 ± 13.1	53.7 ± 12.8	NS
Sex			
Female (%)	42.5	23.8	.003
Male (%)	57.5	76.3	
Body Mass Index (Kg/m^2^)	29.5 ± 5.6	29.9 ± 5.3	NS
Normal (%)	16	20.7	NS
Overweight (%)	32.1	48.3	
Obese (%)	51.9	51.7	
Hypoglycaemic therapies			
Oral antidiabetic drugs (%)	67.9	0	.005
Insulin (%)	9.8	92.5	
Anti‐hypertensive drugs			
ACE inhibitors (%)	47.6	52.0	NS
ARBs (%)	40.5	44.0	
Thiazide diuretics (%)	11.9	24.0	
Others (%)	21.4	28.0	
Hypertension (%)	27.5	31.25	NS
Glucose (mg/dl)	162.5 (133.7–277.2)	175 (132–251)	NS
Urea (mg/dl)	31.5 (18–48.6)	41.3 (25.8–74)	.002
Creatinine (mg/dl)	0.99 (0.7–1.2)	1.1 (0.8–1.7)	.005
Alanine aminotransferase (U/L)	40 (25–65)	37 (29–61)	NS
Aspartate aminotransferase (U/L)	45.5 (26.2–68)	37 (23–49)	.02
Neutrophil count (×10^9^/L)	7.22 (5–24–10.53)	8.7 (6.6–13.5)	.5
Lymphocyte count (×10^9^/L)	0.76(0.5–1.1)	0.76 (0.55–1)	NS
NLR	8.4 (5.5–14.6)	10.8 (6.8–21.4)	.03
Platelet count (×10^9^/L)	191 (156–264)	236.5 (168.5–298.5)	NS
Length of hospital stay (days)	5 (4–9)	7 (4–13)	.03
Fibrinogen (mg/dl)	647.8 ± 201.9	649.1 ± 219.9	NS
D‐dimer (ng/ml)	1229 (766–2788)	1862 (1052–3908)	.009
Distribution of ABO Group			
O (*n*,%)	61, 62.2%	49, 61.2%	NS
A (*n*,%)	32, 32.7%	24, 30.0%	
B (*n*,%)	4, 4.08%	7, 8.8%	
AB (*n*,%)	1, 1.02%	0, 0%	

Note

Parametric variables are represented as mean ± standard deviation. Non‐parametric variables are represented as median and interquartile range (IQR).

Abbreviations: NLR, Neutrophil‐Lymphocyte Count Ratio; NS, No significant.

Patients with hyperglycaemia in the group with IMV received insulin therapy more frequently than patients without VMI (Table 2). Of the patients with admission hyperglycaemia, in the group without IMV, 27.5% were hypertensive; in contrast, in the group of critical patients that required IMV, 31.5% showed hypertension, without significant differences (Table 2). Between these groups, no significant differences were found in hypertension treatment: 47.6% vs. 52% used ACE inhibitors; 40.5% vs. 44% ARBs, other hypertensives 21.4% vs. 28%, respectively.

### 3.3 Association between COVID‐19 severity, mortality and diabetes

In this study, a positive association was found between the severity of the disease, assessed by the need for IMV with admission hyperglycaemia (*p *= .009), and sex (*p *= .04). No association of IMV was found with arterial hypertension, nor with obesity.

D‐dimer level, another marker of poor prognosis of COVID‐19, showed correlation with glucose level (*r* = .154, *p *= .005), creatinine level (*r* = .222, *p *= .0001), platelet count (*r* = .140, *p *= .001), NLR (*r* = .139, *p *= .002) and age (*r* = .125, *p *= .0001).

Mortality in all patients was 47.2% (*n *= 227); 56.2% in the group of patients with admission hyperglycaemia and 38.8% in non‐hyperglycaemia patients.

**FIGURE 2:**
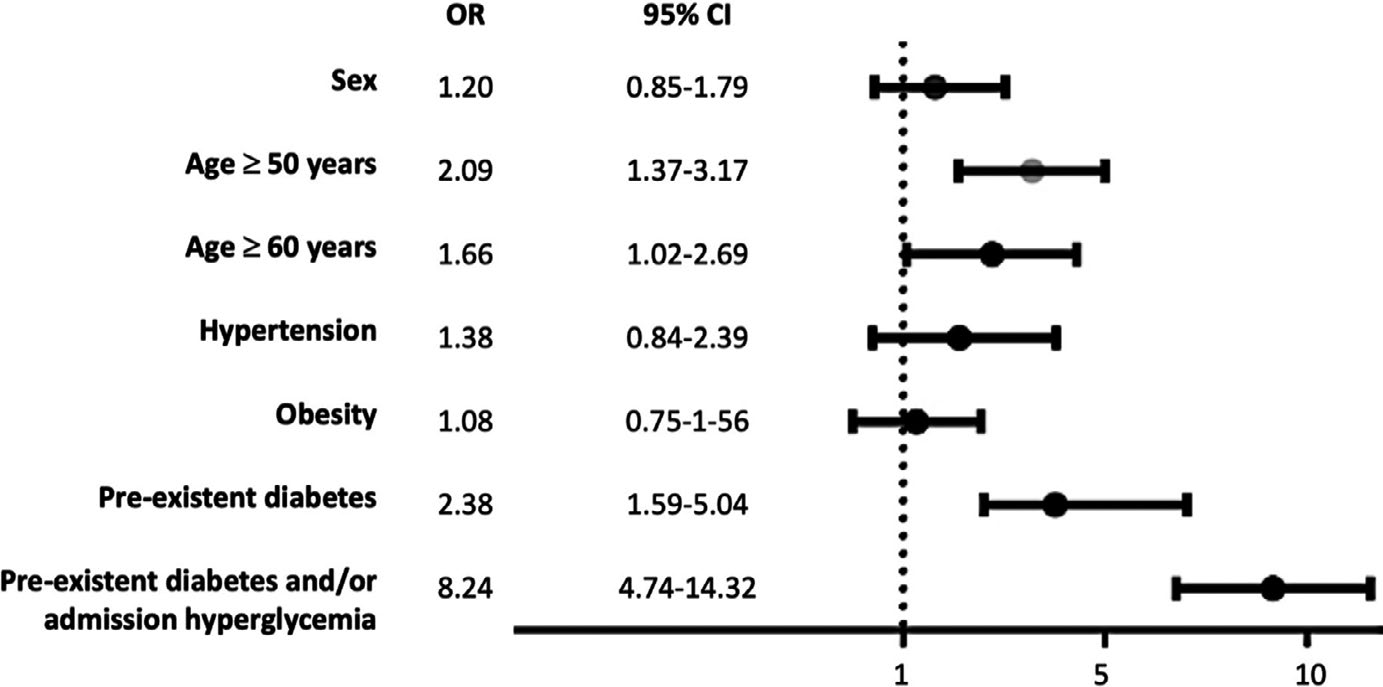
Forest Plot using association of risk factors for mortality, by Odds Ratio and 95% of confidence interval (OR, 95%CI)

It was demonstrated that an age ≥50 years (OR 2.09; 95%CI 1.37–3.17), pre‐existing diabetes (OR 2.38; 95%CI 1.59–5.04) and admission hyperglycaemia (OR 8.24; 95%CI 4.74–14.32) were risk factors for mortality in COVID‐19. There was no evidence of obesity and hypertension as risk factors for mortality in our series (Figure 2).

## 4 DISCUSSION

In this multi‐centre study, we observed the elevated frequency of diabetes and admission hyperglycaemia, and their association with the severity of disease and mortality, in Mexican COVID‐19 patients.

The pre‐existence of certain comorbidities, such as diabetes, has been known as a risk factor for fatality hazard.^3^, ^7^ In our series, at hospital admission of COVID‐19 patients, we noted a high frequency of diabetes (29%); however, when we evaluated the presence of hyperglycaemia, independently of the pre‐existence diagnosis of diabetes, the frequency increased to 48.5%, which is an alarming datum.

Hyperglycaemia has been reported in more than a third of patients admitted to a hospital and is considered an important marker of poor clinical outcomes and increased mortality, not only in critical patients admitted to the intensive care unit, but also in patients admitted to general medicine and surgery wards, in particular in patients without a history of diabetes. Patients with new hyperglycaemia during hospitalization had a significantly higher mortality rate and lower functional outcomes than patients with a known history of diabetes or normoglycaemia.^6^

A proposed mechanism for the increased morbidity and mortality is the effect of hyperglycaemia on different components of the host response, such as the function of immune cells and regulation of cytokines.^17^

Previous studies in patients with severe respiratory syndrome (SARS) have shown that diabetes and hyperglycaemia during the early course of illness were independent predictors for both mortality and morbidity. Their findings raised the possibility that hyperglycaemia might reflect the severity of the viral infection and highlighted the high risk of death and severe hypoxia among diabetic patients developing SARS.^18^

During the COVID‐19 pandemic, these findings continue to be constant. In a retrospective study, Bode et al. reported that patients with diabetes and/or uncontrolled hyperglycaemia were significantly older, had higher HbA1C at 8.7%, higher rate of admission hyperglycaemia, poor glycaemic control during hospitalization, higher rate of hypoglycaemia, longer length of stay and a higher mortality rate.^19^

In another study, Li et al.^20^ reported that hospitalized COVID‐19 patients with newly diagnosed diabetes had the highest risk of all‐cause mortality compared with patients with known diabetes, hyperglycaemia and normal glucose. They proposed as a possible explanation of their findings the suggestion that the upregulated ACE2 receptor expression in cells during acute hyperglycaemia might facilitate viral cell entry.

In a meta‐analysis, Mantovani et al.^21^ observed that at hospital admission, a 14.3% of COVID‐19 patients had pre‐existing diabetes, which is significantly associated with a two‐ to threefold greater risk of severe/critical illness and in‐hospital mortality associated with COVID‐19.

Iacobellis et al.^8^ found that admission hyperglycaemia was the best predictor of radiographic imaging of SARS‐CoV2, regardless of the past medical history of diabetes. They propose that acute hyperglycaemia may lead to an abnormal inflammatory and immune response contributing to the development and progression of the radiographic findings of ARDS in patients with COVID‐19. Likewise, the researchers showed that most of the patients with diabetes who were on ambulatory treatment with DPP4 inhibitors had no radiological findings of ARSD, proposing a potential role of DPP4 in COVID‐19.^8^ Hyperglycaemia remains a strong prognostic predictor of outcome in hospitalized COVID‐19 patients; hyperglycaemia patients display a higher cumulative incidence of severe disease, and glycaemic control is associated with better outcomes.^22^ Moreover, there was evidence that the decrease in glucose levels between baseline and 24 h was associated with a lower rate of progression to severe disease and death at 20 days in both non‐diabetic and diabetic hyperglycaemic patients.^23^

Furthermore, hyperglycaemic crisis, which includes diabetic ketoacidosis (DKA), hyperglycaemic hyperosmolar syndrome (HHS) and combined syndrome of DKA and HHS (DKA/HHS), has also been reported in patients with COVID.^24^ A meta‐analysis revealed that hyperglycaemic crisis is related with a high mortality.^25^ In that study, patients with COVID‐19 and DKA had a mortality rate of 28.9% (95%CI 13.4%–47.5%), but when there was mixed DKA/HHS, mortality reached 31.5% (95%CI 1.2%–78.0%). The mechanisms associated between hyperglycaemic crisis and worse outcomes in COVID‐19 remain uncertain; however, the pro‐inflammatory state observed both COVID‐19 and hyperglycaemic crisis could synergize to lead to worse clinical outcomes.^26^

In a study extracted from an open‐source data set, the General Directorate of Epidemiology of the Mexican Ministry of Health evaluated suspected and confirmed COVID‐19 cases, demonstrating that diabetes, particularly early‐onset diabetes, obesity, COPD, advanced age, immunosuppression and chronic kidney disease were risk factors for lethality.^13^ They reported that early‐onset diabetes conferred an increased risk of hospitalization and a higher risk of mortality in younger patients, which was similar to older patients with comorbidities and only exceeded by older patients with diabetes. The co‐existence of obesity and diabetes, particularly early‐onset diabetes, was a risk factor for COVID‐19 mortality in Mexicans. In addition to diabetes and obesity, other factors associated with lethality were age >65 years, immunosuppression and hypertension.^13^

In contrast, our study was performed in a clinical setting and included only COVID‐ 19 patients that required hospitalization. It was observed that diabetes and admission hyperglycaemia were associated with a higher severity of disease, a worst outcome as the requirement of IMV, and risk of death, considering for both only the history of diabetes or diabetes and/or hyperglycaemia. We observed that an age higher than 60 years was a risk factor for mortality, risk that increased when a cut‐off point of 50 years of age was considered. There was no evidence of an association between obesity and hypertension with the requirement of IMV or COVID‐19 mortality; however, we highlight the increased frequency of hypertension, diabetes and obesity/overweight compared with the general population (hypertension 24% vs. 18.4%; diabetes 29% vs. 10.3%, and obesity/overweight 82.8% vs. 75.2%), according to the National Health Survey in Mexico.^27^

Endothelial dysfunction is a major determinant of COVID‐19, and it is a common feature of the clinical manifestations in patients. SARS‐CoV‐2 uses the surface glycoprotein known as spike to access host cells, and ACE2 is the co‐receptor for coronavirus entry. The density of ACE2 in each tissue may correlate with the severity of COVID‐19. The transmembrane serine protease 2 (TMPRSS2), sialic acid receptors and extracellular matrix metalloproteinase inducer (CD147 or basigin) are other receptors on the surface of human cells that mediate the entry of SARS‐CoV‐2. All of these factors are expressed in endothelial cells. Endothelial dysfunction is aggravated by hypercoagulation and hypoxia, which augments thrombosis, embolism and disseminated intravascular coagulation.^4^ In this setting, TMPRSS2 represents a valid target in COVID‐19 treatment and miR‐98‐5p is a suitable candidate; miR‐98 directly targets the 3′UTR of TMPRSS, having a key role in the regulation of endothelial function.^28^

In the present study, patients with admission hyperglycaemia exhibited lower lymphocyte count and increased NLR and D‐dimer, compared to non‐hyperglycaemia patients. D‐dimer originates from the formation and lysis of cross‐linked fibrin and reflects activation of coagulation and fibrinolysis. D‐dimer level on admission has been considered an independent predictor for in‐hospital mortality in patients with COVID‐19.^29^ It has been proposed that hypercoagulable state in COVID‐19 could be related with an aggressive pro‐inflammatory response, endothelial dysfunction, pro‐thrombosis conditions such as being bedridden, sepsis‐induced coagulopathy and/or disseminated intravascular coagulation.^30^

Guo et al.^30^ reported that patients with COVID‐19 and diabetes had a more severe SARS‐CoV‐2 pneumonia, D‐dimer, ferritin, fibrinogen and absolute neutrophils, as well as lymphopenia and lower haemoglobin, showing that these patients were at higher risk of excessive uncontrolled inflammation response and hypercoagulable state, which may have contributed to a poorer prognosis of COVID‐19.

Yan et al.^31^ showed that patients with COVID‐19 and diabetes had more comorbidities such as cardiovascular, cerebrovascular disease or hypertension, had a longer length of hospital stay, were more likely to receive mechanical ventilation and admission to ICU, and had a higher mortality. Biochemically, these patients had higher levels of leucocyte count, neutrophil count, hsCRP, ferritin, IL‐2 receptor, IL‐6, IL‐8, TNFα, D‐dimer, fibrinogen and N‐terminal pro‐brain natriuretic peptide (NT‐proBNP).

On the other hand, a possible relation of specific blood group and the association with an increased risk of SARS‐CoV‐2 infection has been proposed. A meta‐analysis demonstrated that blood type A might be more susceptible to SARS‐CoV‐2 infection, while blood type O might be less susceptible; there was no correlation between ABO blood group and severity or demise of COVID‐19.^32^

In patients of the present study, no ABO group differences were observed. However, the initial objective was not to study the association between blood groups and COVID‐19. In Mexico, the distribution of blood types varies compared with other populations, so that group O predominates and the proportion of the population with group A is lower.^33^ More studies focussed on the relation between ABO group and clinical outcome are required.

The limitations of our study include the retrospective design and the lack of resources for biochemical evaluation of parameters such as HbA1c or interleukin profile which is a non‐routine measurement in some health centres due to economic limitations. The main strengths of this study are its conduction in a clinical setting and the evaluation of outcomes not yet explored in a low/middle‐income country population, where there is a high prevalence of cardiometabolic diseases.

The present study denotes the devastating conjunction of hyperglycaemia and COVID‐19. Mexico is one of the countries with the highest prevalence of diabetes in the world and is currently also one of the countries with the highest mortality from COVID‐19: as of May 23, 2021, 221 647 had died, according to official statistics.

The findings in our study highlight the importance of the detection of hyperglycaemia in patients with COVID‐19, both with and without previous diagnosis of diabetes, and the timely treatment and restoration of normoglycaemia, which could improve the prognosis. We consider that, now more than ever, priority must be placed on the emphasis on an adequate glycaemic control in patients with diabetes; likewise, on compliance and adherence to non‐pharmacological and pharmacological treatment in patients with any alteration of carbohydrate metabolism. The timely detection and medical evaluation of COVID‐19 symptoms in this vulnerable population, the importance of the screening for metabolic alterations in patients with COVID‐19 since the beginning of the disease, and the promotion of healthy lifestyles in general population despite the limitations stemming from the pandemic, are essential.

## CONFLICT OF INTEREST

The authors declare no conflict of interest in this study.

## AUTHOR CONTRIBUTIONS

All authors participated in the research and article preparation. CMM and CRP conceived and designed the analysis; CMM, CRP, KPT, EF, BL, EM, GB and JFZ collected the data; MBR, CMM, CRP, KPT, LB and LBH contributed data or analysis tools; LB, LBH and RRT, MSL performed the analysis; LB, LBH and CMM wrote the paper. All authors approved the final article.

## ETHICAL STATEMENT

The study was approved by the local Ethical Committee of General Hospital of Mexico, Mexico City, Mexico.

## PROTECTION OF HUMAN SUBJECTS

The authors declare that no experiments were performed on humans for this study. Confidentiality of data. The authors declare that no personal information appears in this manuscript. Manuscript contains original, unpublished work that is not being considered for publication elsewhere.

## Data Availability

NA.
